# Exposure-response relationship of sertraline in pediatric patients with anxiety disorders: a population pharmacokinetic analysis

**DOI:** 10.3389/fphar.2026.1862275

**Published:** 2026-06-24

**Authors:** Jesus Alonso Gandara Mireles, Julio Cesar Grijalva Avila, Ignacio Villanueva Fierro, Cynthia Mora Muñoz, Pedro Sanchez Campos, Sagrario Salas Name, Maura Antonia Lazcano Franco, Adrián Arceo Durán, Karla Patricia Rosales Silva, Hugo Ontiveros Sánchez, Maria Guadalupe Nieto Pescador, Odín Romero Soto, Verónica Loera Castañeda, Leslie Patrón Romero, Horacio Almanza Reyes

**Affiliations:** 1 CIIDIR-Durango Unit, National Polytechnic Institute, Genomics Academy, Durango, Mexico; 2 Latin American Network for the Implementation and Validation of Pharmacogenomic Clinical Guidelines (RELIVAF-EMERITA), Santiago, Chile; 3 Faculty of Psychology and Human Communication Therapy, Juárez University of the State of Durango, Durango, Mexico; 4 Durango State Cancer Center (CECAN), Durango, Mexico; 5 General Hospital of Durango, Maternal-Child Tower, Durango, Mexico; 6 Faculty of Medicine and Nutrition (FAMEN), Universidad Juárez del Estado de Durango, Durango, Mexico; 7 Durango Mental Health Hospital, SSD, Durango, Mexico; 8 Faculty of Chemical Sciences, Juárez University of the State of Durango, Durango, Mexico; 9 School of Medicine and Psychology, Autonomous University of Baja California, Tijuana, Mexico

**Keywords:** adverse events, anxiety, pediatrics, pharmacokinetics/pharmacodynamics, sertraline

## Abstract

**Background:**

Sertraline is widely used for the treatment of anxiety disorders in pediatric populations; however, substantial interindividual pharmacokinetic (PK) variability may significantly influence therapeutic efficacy and the occurrence of adverse events (AEs).

**Objective:**

To characterize the population PK profile of sertraline in pediatric patients with anxiety disorders and to evaluate its association with clinical response and AEs.

**Methods:**

A prospective population PK study was conducted in 85 pediatric patients with anxiety disorders treated with sertraline monotherapy. Plasma concentrations were quantified using high-performance liquid chromatography with fluorescence detection. Population PK modeling was performed using nonlinear mixed-effects models (Monolix®). Anxiety severity was assessed using the Generalized Anxiety Disorder-7 (GAD-7) scale, and AEs were recorded throughout follow-up.

**Results:**

The mean dose was 35 ± 15 mg/day. High interindividual variability was observed in average plasma concentrations. Overall, 30.5% of patients experienced at least one adverse event, with nausea and insomnia being the most frequent. Patients with AEs exhibited lower apparent clearance and central volume of distribution, along with higher peak concentrations. Conversely, patients with greater anxiety severity showed higher clearance and lower steady-state average concentrations. An interquartile range of average concentrations (Cavg) between 27.85 and 57.91 ng/mL was associated with a favorable balance between efficacy and safety.

**Conclusion:**

PK variability of sertraline is associated with differences in clinical response and AEs in pediatric patients. These findings suggest that systemic exposure to sertraline may be associated with clinical response and AEs, supporting the potential role of therapeutic drug monitoring and dose individualization.

## Introduction

Anxiety disorders are among the most prevalent psychiatric conditions in pediatric populations, with an estimated incidence ranging from 6% to 20% in children and adolescents worldwide (Merikangas et al., 2010; Polanczyk et al., 2015). These disorders not only impair emotional and social functioning but are also associated with an increased risk of psychiatric disorders in adulthood, functional impairment, and reduced quality of life (Pine et al., 1998). In this context, pharmacological treatment represents a critical therapeutic strategy when psychotherapeutic interventions are insufficient or in cases of greater clinical severity (Walkup et al., 2008). Selective serotonin reuptake inhibitors (SSRIs) are considered first-line pharmacological agents for pediatric anxiety disorders, with sertraline being one of the most frequently prescribed due to its favorable efficacy and tolerability profile (Strawn et al., 2015; Ipser et al., 2009). Its mechanism of action involves inhibition of serotonin reuptake at the synaptic level, increasing its availability in the central nervous system and modulating neural circuits involved in mood and anxiety regulation (Stahl, 1998). Clinical trials have demonstrated its efficacy in generalized anxiety disorder, obsessive-compulsive disorder, and related conditions in pediatric populations (Rynn et al., 2001; Wagner et al., 2003).

Despite its widespread use, the clinical response to sertraline shows marked interindividual variability in both efficacy and the occurrence of adverse events (AEs) (Preskorn, 1997; Bridge et al., 2007). While some patients achieve significant symptom improvement, others exhibit partial response or develop AEs that may limit treatment adherence. This variability may be partially explained by multiple factors, including demographic, physiological, and genetic characteristics, as well as differences in pharmacokinetic processes and exposure-response relationships (Stingl et al., 2013; Kirchheiner et al., 2004). From a pharmacokinetic (PK) perspective, sertraline is characterized by relatively slow oral absorption, high lipophilicity, extensive plasma protein binding, and significant hepatic metabolism mediated primarily by cytochrome P450 enzymes, including CYP2C19, CYP2B6, and CYP2D6 (Obach et al., 2005; Li et al., 2025). These metabolic pathways exhibit substantial interindividual variability, which may contribute to differences in clearance, volume of distribution, and systemic exposure (Mandrioli et al., 2013). However, pharmacogenetic factors were not directly evaluated in the present study, and therefore the contribution of specific CYP phenotypes could not be assessed within this cohort. In pediatric populations, this variability may be further amplified by developmental changes in physiology and enzyme activity (Kearns et al., 2003). Sertraline is predominantly metabolized into N-desmethylsertraline, its major circulating metabolite, which has considerably lower pharmacological activity than the parent compound (Wang et al., 2008).

From a pharmacodynamic (PD) standpoint, the relationship between sertraline plasma concentrations and clinical effects also exhibits considerable variability. Emax-type PD models have been used to describe the relationship between drug exposure and therapeutic response or AEs in different populations (Wang et al., 2008; Holford and Sheiner, 1981). However, integrated characterization of exposure-response relationships in pediatric patients remains limited. In recent years, integrated pharmacokinetic/pharmacodynamic (PK/PD) approaches have emerged as essential tools for understanding the relationship between drug exposure and clinical outcomes. These approaches enable the identification of patient subgroups at higher risk of toxicity or reduced therapeutic response, supporting the development of individualized dosing strategies (Mould and Upton, 2012). Nevertheless, studies integrating PK/PD data for sertraline in pediatric populations, particularly in Latin American settings, remain scarce (Strawn et al., 2022). Although recent studies have demonstrated substantial interindividual variability in sertraline metabolism in pediatric patients (Poweleit et al., 2023; Baumann et al., 2004), further research is needed to directly link systemic exposure with clinical response and AEs.

In this context, the identification of exploratory exposure-response associations may contribute to the development of individualized therapeutic strategies, optimizing clinical efficacy while minimizing AEs. Accordingly, the present study aimed to evaluate the association between sertraline exposure, characterized through population PK parameters, and clinical outcomes, including anxiety severity and the occurrence of AEs, in a pediatric cohort with anxiety disorders.

## Materials and methods

### Study design

A prospective population pharmacokinetic (PKpop) study was conducted to evaluate the association between systemic exposure to sertraline, clinical response (measured as anxiety severity), and the occurrence of AEs in pediatric patients with anxiety disorders. A total of 85 pediatric patients diagnosed with anxiety disorders and treated with sertraline monotherapy were enrolled from two clinical centers in Mexico: the Pediatric Service of the Mental Health Hospital of the Ministry of Health in Durango and the AMCII Specialty Hospital in Durango. Patients with severe comorbidities or those receiving concomitant medications with potential to interfere with sertraline pharmacokinetics were excluded. Participants were followed for 6 months from treatment initiation.

The diagnosis of anxiety disorders was established by child and adolescent psychiatry specialists according to the *Diagnostic and Statistical Manual of Mental Disorders, Fifth Edition* (DSM-5), using structured clinical assessment (American Psychiatric Association, 2013). Anxiety severity was assessed using the *Generalized Anxiety Disorder-7* (GAD-7) scale (Spitzer et al., 2006; Löwe et al., 2008), administered at baseline and at the end of follow-up.

### Safety assessment

AEs were prospectively recorded during clinical follow-up through patient-reported symptoms and physician assessments. All events were systematically documented at each clinical visit and recorded in the medical records by the treating physician. Follow-up visits were conducted approximately every 2 weeks, and patients and caregivers were actively questioned regarding the occurrence of treatment-emergent AEs. Event severity was graded according to the Common Terminology Criteria for Adverse Events (CTCAE). All reported AEs occurred within the first month of sertraline treatment.

### Ethical considerations

The study was approved by the Research Ethics Committee and Research Committee of Hospital General 450, Durango, Mexico (approval numbers CEI-HG450-24/149 and CI-HG450-24/149), and conducted in accordance with the Declaration of Helsinki and the Mexican General Health Law. Written informed consent was obtained from parents or legal guardians, along with assent from patients older than 7 years.

### Pharmacological treatment

All patients received sertraline in monotherapy as part of routine clinical practice, following international guidelines for pediatric anxiety disorders. Treatment was initiated at 25 mg/day, with subsequent dose adjustments individualized based on clinical response and tolerability (Huddart et al., 2020; Hussain et al., 2016).

### Biological sample collection

Peripheral blood samples were collected for sertraline quantification to characterize its PKpop profile. Given the clinical context and pediatric population, a sparse sampling design was implemented, allowing robust estimation of individual and PKpop parameters using nonlinear mixed-effects modeling (Joshi et al., 2023). Each patient contributed between one and three samples, randomly assigned across the dosing interval to capture absorption, distribution, and elimination phases. Sampling times included predose (0 h) and 1–25 h post-dose (1–25 h), distributed randomly across individuals (Tam et al., 2003). Blood samples were collected in anticoagulant tubes and centrifuged at 3500 rpm for 10 min at 4 °C to separate plasma. Plasma samples were transferred to microtubes and stored at −80 °C until analysis. The time from sample collection to processing was maintained below 30 min to ensure analyte stability.

Pharmacokinetic sampling was performed under presumed steady-state conditions. Patients were required to have received sertraline for at least five elimination half-lives prior to sample collection, corresponding to approximately 5 days of continuous treatment. Medication adherence was assessed during routine clinical visits through interviews with parents or primary caregivers. For each observation, the exact time of the most recent dose administration and the exact time of blood sample collection were recorded and incorporated into the pharmacokinetic dataset used for model development.

### Analytical quantification

Plasma concentrations of sertraline were determined using high-performance liquid chromatography (HPLC) with fluorescence detection. Analyses were performed using an Agilent 1100 system (Agilent Technologies, United States) equipped with a fluorescence detector. Chromatographic separation was achieved on a reverse-phase C18 column (150 mm × 4.6 mm, 5 µm particle size; Thermo Fisher Scientific, United States) under isocratic conditions. The mobile phase consisted of a phosphate buffer and acetonitrile mixture (33:67, v/v), providing adequate resolution of analytes.

Plasma samples underwent protein precipitation using methanol prior to analysis to remove interfering substances and improve analyte recovery. Calibration curves were constructed over a concentration range of 5–200 ng/mL, with a lower limit of quantification (LLOQ) of 2 ng/mL. Concentrations below the lower limit of quantification (LLOQ) were retained in the analysis and treated as censored observations below the quantification limit rather than being excluded or replaced by fixed values. Correlation coefficients (r^2^) exceeded 0.99. Method precision and accuracy were evaluated according to internationally accepted bioanalytical validation criteria. The method was validated in accordance with the Mexican Official Standard NOM-177-SSA1-2013 (NORMA, 2013), ensuring reliable quantification of sertraline within the studied concentration range.

### Population pharmacokinetic modeling

PKpop analysis was performed using a nonlinear mixed-effects approach implemented in Monolix® (Lixoft, France). An initial base model without covariates was developed to characterize structural and interindividual variability. Different structural models were evaluated, including one- and two-compartment models with first-order absorption, with or without an absorption lag time (Tlag). Model selection was based on statistical criteria, including reductions in objective function value (OFV), Akaike information criterion (AIC), and Bayesian information criterion (BIC), as well as goodness-of-fit diagnostics and biological plausibility. Given the sparse pediatric sampling design, structural model selection was based not only on statistical criteria but also on diagnostic performance, predictive capability, bootstrap robustness, and biological plausibility. One-compartment and two-compartment models with first-order absorption, with and without absorption lag time, were sequentially evaluated using nonlinear mixed-effects modeling.

Parameter estimation was conducted using the stochastic approximation expectation-maximization (SAEM) algorithm, allowing robust estimation under sparse data conditions. Interindividual variability was modeled assuming a log-normal distribution. Residual variability was evaluated using additive, proportional, and combined error models, selecting the structure that best described unexplained variability. PK parameters were scaled according to body surface area using linear normalization, with a reference value of 1.24 m^2^.

### Covariate analysis

To explain interindividual variability, the influence of demographic and clinical covariates on PK parameters was evaluated using a stepwise covariate modeling approach. Covariates were first screened in the base model and subsequently incorporated based on statistical significance and biological plausibility. Covariate inclusion required a significance level of p < 0.01, while retention during backward elimination required p < 0.001. Evaluated covariates included age, body weight, sex, administered dose, anxiety severity, and presence of AEs.

### Model evaluation

Model development followed a sequential approach: (i) base model identification, (ii) covariate inclusion, and (iii) backward elimination. Model performance was assessed using OFV, AIC, BIC, and parameter precision, expressed as relative standard error (RSE), with values < 15% considered acceptable. Goodness-of-fit plots included observed versus predicted concentrations and individual weighted residuals (IWRES). Model adequacy was confirmed when residuals were randomly distributed without systematic trends. Model robustness was assessed using nonparametric bootstrap analysis (1000 replicates), comparing bootstrap estimates with final model parameters. Predictive performance was evaluated using visual predictive checks (VPCs).

### Visual predictive check (VPC)

A visual predictive check was performed to evaluate the predictive performance of the model. Simulated datasets were generated from the final model, and the 5th, 50th, and 95th percentiles were compared with observed data. Model adequacy was confirmed when observed percentiles fell within the 90% prediction intervals derived from simulations.

### Exposure-response analysis

The exposure-response relationship was evaluated using anxiety severity (*GAD-7* score) as the PD endpoint. Patients were initially classified into four severity categories and subsequently grouped into two clinically relevant subgroups: minimal/mild and moderate/moderately severe anxiety. Steady-state average concentrations (Cavg) were compared between groups. Additionally, associations between individual PK parameters (particularly apparent clearance, CL/F) and clinical outcomes were explored. The relationship between exposure and AEs was initially evaluated using univariate logistic regression models. Additional multivariable logistic regression analyses adjusting for age, BMI, and sertraline dose were subsequently performed as sensitivity analyses to assess the robustness of the observed associations. The dependent variable was the presence of clinically relevant AEs, while independent variables included CL/F and Cmax. CL/F was categorized (high vs. low) using the population mean as a pragmatic cutoff to facilitate clinical interpretability. This approach was chosen for clinical interpretability, although it may reduce information compared with continuous modeling.

### Parameter estimation

PK parameters were estimated using the SAEM algorithm in Monolix®. Parameter precision was evaluated using relative standard error (RSE) derived from linearization methods. This approach provided robust population and individual parameter estimates.

### Exposure metrics and therapeutic range derivation

Individual *post hoc* parameters were used to derive exposure metrics, including Cavg, calculated as AUC_0_-τ/τ, where τ represents the dosing interval. For each patient, steady-state simulations were performed using the individual *post hoc* Bayesian parameter estimates obtained from the final population pharmacokinetic model together with the recorded dosing regimen. AUC_0_-τ was calculated from the simulated concentration-time profiles and subsequently used to derive Cavg. Only observations with complete dosing information were included in the simulations. The area under the curve (AUC) was obtained through steady-state simulations based on the final PK model. AUC was derived from model-based simulations and used exclusively for the calculation of Cavg; it was not analyzed as an independent pharmacokinetic endpoint. An empirical therapeutic range was derived from patients with favorable clinical response (minimal/mild anxiety) and absence of AEs. Percentiles and interquartile ranges were used to identify an exposure window associated with optimal efficacy and safety. The exposure range associated with favorable clinical outcomes was derived exclusively from individual *post hoc* Cavg estimated from the final population pharmacokinetic model. Specifically, the interquartile range (IQR; 25th-75th percentiles) was calculated using data from patients who achieved minimal or mild anxiety severity (GAD-7) and did not experience AEs. This subgroup was selected to represent individuals with an optimal balance between therapeutic efficacy and tolerability. Therefore, the proposed exposure range reflects the distribution of Cavg values in this clinically favorable subgroup rather than the entire cohort.

### Statistical analysis

Data are presented as mean ± standard deviation or proportions, as appropriate. Normality of continuous variables was assessed using graphical and statistical methods. Between-group comparisons were performed using Student’s t-test for continuous variables and chi-square or Fisher’s exact test for categorical variables. Associations between PK parameters and AEs were evaluated using logistic regression models, reporting odds ratios (ORs) with 95% confidence intervals. Statistical significance was defined as p < 0.05. Analyses were performed using R software (R Foundation for Statistical Computing, Vienna, Austria).

## Results

### Population characteristics and pharmacokinetic data

**FIGURE 1:**
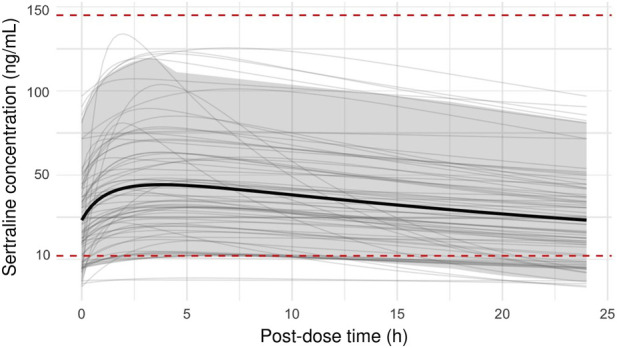
Sertraline concentration-time profile in pediatric patients with anxiety disorders. Gray lines represent individual observed plasma concentrations, while the solid black line corresponds to the population pharmacokinetic model prediction.

A total of 85 pediatric patients with anxiety disorders treated with sertraline monotherapy were included in the study. Among the cohort, 59 patients did not experience AEs, whereas 26 patients (30.5%) developed at least one AE. Baseline characteristics were generally comparable between groups. Mean age was similar in patients with and without AEs (12.22 vs. 12.08 years, respectively). Differences were observed in body weight, which was lower in patients without AEs (47.76 kg) compared with those who experienced AEs (51.87 kg). A trend toward higher body mass index (BMI) values was also observed in patients with AEs (23.9 ± 4.2 vs. 21.2 ± 4.5 kg/m^2^), reaching borderline statistical significance (p = 0.046). Detailed baseline characteristics are presented in Table 1. Each patient contributed between one and three samples according to the population sampling design, resulting in a total of 218 plasma concentrations used for PKpop model development. Concentration-time profiles showed substantial interindividual variability; however, the population mean profile adequately described the central tendency of the data, characterized by a rapid increase following drug administration followed by a gradual elimination phase. Most individual profiles fell within the therapeutic range reported in the literature (10–150 ng/mL) (Figure 1).

**TABLE 1:** Demographic and clinical characteristics of pediatric patients with and without AEs.

Variable	No AEs (n = 59)	AEs (n = 26)	*p value*
Age (years)	12.22 ± 4.01	12.08 ± 3.99	0.78*
Weight (kg)	47.76 ± 15.02	51.87 ± 13.22	0.06*
Height (m)	1.50 ± 0.10	1.49 ± 0.10	0.32*
BMI (kg/m^2^)	21.2 ± 4.5	23.9 ± 4.2	0.04*
Dose (mg/day)	35 ± 15.00	35 ± 15.00	1.00*
Sex (M/F)	32/27	16/10	0.65**

Data are presented as mean ± standard deviation unless otherwise indicated.

AEs: adverse events; Kg: Kilograms; m: meters; BMI: body mass index; mg: milligrams; M: male; F: female; *: *p* values calculated using Student’s t-test for independent samples; **: *p* value calculated using the chi-square test.

### Population pharmacokinetic model development and validation

The PKpop model that best described the data was a two-compartment model with first-order absorption, an absorption lag time (Tlag), and first-order elimination. Compared with the initial one-compartment model, the final two-compartment model with lag time showed substantial improvement in statistical performance, with reductions in OFV from 2317 to 2216, AIC from 1298 to 982, and BIC from 1332 to 1044. In addition, the final model demonstrated improved goodness-of-fit diagnostics, more adequate characterization of the concentration decline phase, random distribution of residuals without systematic trends, acceptable visual predictive check performance, and stable bootstrap behavior. In the covariate analysis, body weight and BMI were identified as significant predictors of apparent clearance (CL/F) and central volume of distribution (V1/F), reducing unexplained interindividual variability and improving overall model performance. Inclusion of body weight and BMI reduced the interindividual variability of CL/F from 38% to 28% (26.3% reduction) and V1/F from 48% to 35% (27.1% reduction). A smaller reduction was also observed for Ka (31%–25%; 19.4% reduction). The magnitude and direction of covariate effects are summarized in Supplementary Figure S1. The final parameter estimates were as follows: CL/F = 59.7 L/h, V1/F = 1200 L, V2/F = 1350 L, and Q/F = 161 L/h. The absorption rate constant (Ka) was 0.7 h^-1^ and the lag time (Tlag) was 0.30 h. Because sertraline was administered orally, all clearance and volume parameters are reported as apparent values (CL/F, V1/F, V2/F, and Q/F). Therefore, V1/F and V2/F should not be interpreted as true physiological volumes, since absolute oral bioavailability was not independently estimated. Additional *post hoc* diagnostic evaluation showed low eta-shrinkage for CL/F (12.4%) and V1/F (18.9%), moderate shrinkage for ka (29.1%), and higher shrinkage for V2/F (43.6%) and Q/F (43.7%). Eta-correlation analysis showed a moderate correlation between ETA_CL and ETA_V1 (r = 0.73), whereas correlations involving peripheral distribution parameters were low, including ETA_V2–ETA_Q (r = 0.10), ETA_V2–ETA_V1 (r = −0.07), and ETA_Q–ETA_CL (r = −0.03). The low eta-shrinkage observed for CL/F and V1/F supports the reliability of individual *post hoc* Bayesian estimates used for the derivation of exposure metrics, including AUC_0_-τ and Cavg.

**FIGURE 2:**
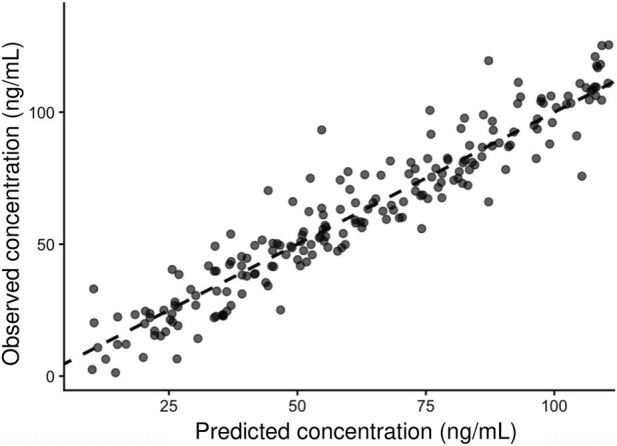
Observed versus predicted sertraline plasma concentrations based on the population pharmacokinetic model. Each point represents an individual observation. The dashed line corresponds to the line of identity (y = x), indicating perfect agreement between observed and predicted concentrations. The distribution of the data around this line suggests an adequate model fit and good predictive performance across the evaluated concentration range.

**FIGURE 3:**
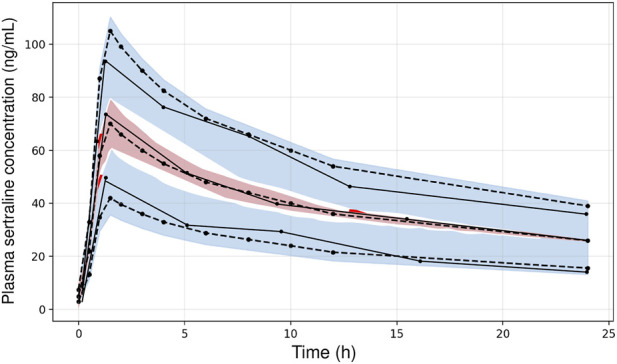
Visual predictive check (VPC) of the sertraline population pharmacokinetic model. Lines represent the observed plasma concentrations over time, while shaded areas correspond to the prediction intervals derived from 1000 model-based simulations. The solid line indicates the median of the observed concentrations. The dashed lines represent the 5th and 95th percentiles of the observed data. The agreement between observed percentiles and model-derived prediction intervals suggests adequate predictive performance in describing both the central tendency and variability of sertraline plasma concentrations in the pediatric population.

Interindividual variability was moderate, with coefficients of variation of 28% for CL/F, 35% for V1/F, and 25% for Ka, indicating clinically relevant heterogeneity in drug disposition. Final model parameters are summarized in Table 2. The model showed adequate goodness-of-fit, evidenced by agreement between observed and predicted concentrations and random distribution of residuals without systematic bias (Figure 2). Visual predictive checks (VPCs) demonstrated good predictive performance, with observed 5th, 50th, and 95th percentiles falling within model-derived prediction intervals (Figure 3). Bootstrap analysis (1000 replicates) further confirmed model robustness, showing high concordance between original and resampled parameter estimates.

**TABLE 2:** Final population pharmacokinetic parameters of sertraline.

Parameter (units)	Description	Base model (RSE, %)	Final model (RSE, %)	Interindividual variability (CV, %)	Bootstrap mean (n = 1000)	95% CI
OFV	Objective function	2317	2216	-	2220	2190–2255
AIC	Akaike	1298	982	-	990	970–1015
CL/F (L/h)	Apparent clearance	59.6 (9.0%)	59.7 (9.0%)	28	60.0	50.5–69.8
V1/F (L)	Central volume of distribution	1205 (12.0%)	1200 (12.0%)	35	1215	980–1490
Q/F (L/h)	Intercompartmental clearance	162 (15.0%)	161 (15.0%)	40	158	120–210
V2/F (L)	Peripheral volume of distribution	1360 (14.0%)	1350 (14.0%)	38	1330	980–1820
Tlag (h)	Absorption lag time	0.30 (22.0%)	0.30 (22.0%)	30	0.31	0.20–0.40
Ka (h^-1^)	Absorption rate constant	0.7 (18.0%)	0.7 (18.0%)	25	0.8	0.17–1.55
Cmax (ng/mL)	Maximum concentration	67.19 (2.79%)	67.19 (2.79%)	32.29	67.26	63.56–70.74
t½ (h)	Elimination half-life	26.06 (18.14%)	27.56 (11.14%)	08.50	26.42	7.56–74.52

	Values		S.E.	RS.E.
Model error	B	0.723	0.0077	7.82
Parameters	a	0.882	0.044	11.93

Population pharmacokinetic parameter estimates are reported with relative standard errors (RSE, %) in parentheses. Interindividual variability is expressed as coefficient of variation (CV, %). Bootstrap results (1000 replicates) are presented as mean parameter estimates with 95% confidence intervals (CI). OFV: objective function value; AIC: akaike information criterion; CL/F: apparent clearance; V1/F: central volume of distribution; V2/F: peripheral volume of distribution; Q/F: intercompartmental clearance; Ka: absorption rate constant; Tlag: lag time; Cmax: maximum concentration; t½(h): elimination half-life; -: not applicable.

### Relationship between pharmacokinetics, AEs, and clinical response

**FIGURE 4:**
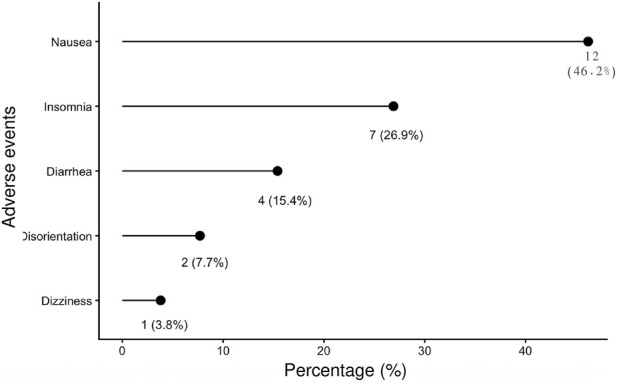
Distribution of AEs in pediatric patients treated with sertraline. Each point represents the proportion of patients experiencing a specific adverse event, with labels indicating absolute counts and corresponding percentages. Nausea and insomnia were the most frequently observed AEs.

Among the 26 patients who experienced AEs, nausea was the most frequently reported (46.2%), followed by insomnia (26.9%). Less frequent events included diarrhea (15.4%), disorientation (7.7%), and dizziness (3.8%). The distribution of AEs is summarized in Figure 4.

Significant differences in PK parameters were observed between patients with and without AEs. Patients who developed AEs exhibited significantly lower apparent clearance (38.4 ± 6.1 vs. 62.7 ± 7.8 L/h; p = 0.01) and lower central volume of distribution (820 ± 95 vs. 1310 ± 410 L; p = 0.03). Additionally, these patients showed higher peak concentrations (Cmax) (78.5 ± 13.4 vs. 42.1 ± 12.8 ng/mL; p = 0.03), indicating increased systemic exposure. Furthermore, 55.6% of patients with AEs had CL/F values below the population mean, compared with 9.5% in patients without AEs. These findings are detailed in Table 3. Regarding clinical response, patients with higher anxiety severity exhibited significantly higher apparent clearance (62.3 ± 9.6 vs. 48.7 ± 11.4 L/h; p = 0.02), accompanied by Cavg (20.1 ± 8.13 vs. 57.4 ± 19.1 ng/mL; p = 0.01). These results suggest that markedly lower systemic exposure to sertraline is associated with increased anxiety severity. Results are presented in Table 4. Univariate logistic regression analysis showed that lower apparent clearance was associated with a higher probability of developing AEs (OR = 3.23; 95% CI: 1.022–13.032; p = 0.04). Similarly, higher Cmax values were associated with increased risk of AEs (OR = 2.11; 95% CI: 1.008–16.109; p = 0.03). Notably, patients who experienced AEs showed higher Cavg values with relatively lower variability compared to those without AEs, suggesting a more homogeneous exposure pattern at higher concentration levels. Additional multivariable logistic regression analyses were performed to evaluate whether the observed exposure-response associations persisted after adjustment for age, BMI, and sertraline dose. The association between higher systemic exposure and AEs remained significant after adjustment. Similarly, the associations between exposure metrics and anxiety severity remained directionally consistent after adjustment. These findings suggest that the observed exposure-response associations were not fully explained by the evaluated covariates, although residual confounding cannot be excluded. Detailed results are provided in Supplementary Table S2.

**TABLE 3:** Comparison of pharmacokinetic parameters between patients with and without AEs.

Group	n	CL/F (L/h)	% low cl/f**	V1/F (L)	% low V1/F**	Cmax (ng/mL)
With AEs	26	38.4 ± 6.1	55.6%	820 ± 95	22.2%	78.5 ± 13.4
Without AEs	59	62.7 ± 7.8	9.5%	1310 ± 410	4.8%	42.1 ± 12.8
p*	-	0.01*	-	0.03*	-	0.02*

Data are presented as mean ± standard deviation unless otherwise indicated. AEs: adverse events; CL/F: apparent clearance; V1/F: central volume of distribution; Cmax: maximum concentration; *: *p* values calculated using Student’s t-test for independent samples; **: Low CL/F defined as values <59.7 L/h (population mean); low V1/F defined as values <1200 L (population mean); —: not applicable.

**TABLE 4:** Relationship between pharmacokinetic parameters and anxiety severity.

Group (*GAD-7*)	n	CL/F (L/h)	Cavg (ng/mL)
All patients			
Moderate/Moderately severe anxiety	36	62.3 ± 9.6	20.1 ± 8.1
Minimal/Mild anxiety	49	48.7 ± 11.4	57.4 ± 19.1
p-value*		0.02*	0.01*
With AEs (n = 26)			
Moderate/Moderately severe anxiety	6	64.7 ± 2.4	25.1 ± 13.1
Minimal/Mild anxiety	20	41.5 ± 3.2	76.6 ± 4.1
p-value*		0.03*	0.01*
Without AEs (n = 59)			
Moderate/Moderately severe anxiety	21	51.1 ± 8.4	27.2 ± 4.1
Minimal/Mild anxiety	38	38.9 ± 8.3	41.2 ± 6.1
p-value*	​	0.04*	0.04*

Data are presented as mean ± standard deviation; CL/F: apparent clearance; Cavg: steady-state average concentration; *: * p-values were calculated using Student’s t-test for independent samples.

### Derivation of exposure range associated with clinical outcomes

**FIGURE 5:**
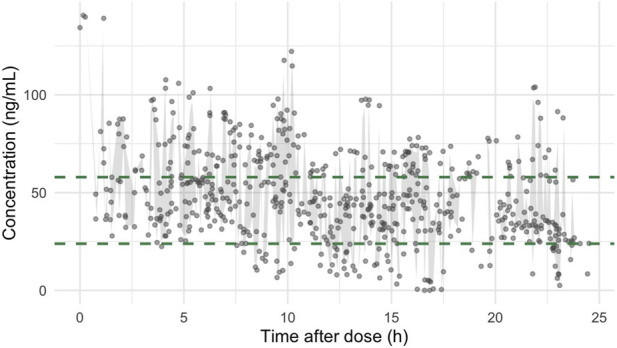
Observed sertraline concentrations in pediatric patients with anxiety disorders. Each point represents an observed plasma concentration, with each patient contributing between one and three samples across the dosing interval. The transparent gray shaded area represents the 5th to 95th percentile range of concentrations (P10-P90), illustrating the variability in systemic exposure within the population. Dashed horizontal lines (green)indicate the proposed exposure range associated with a favorable balance between efficacy and safety (27.85–57.91 ng/mL). The dispersion of observations relative to this range highlights interindividual variability and the proportion of concentrations falling within, below, or above the proposed a preliminary exposure range.

**FIGURE 6:**
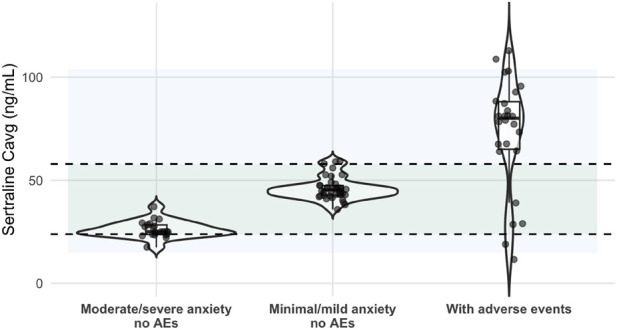
Distribution of steady-state average concentrations (Cavg) according to clinical outcome. Violin plots depict the distribution of steady-state average concentrations (Cavg) in patients with moderate/moderately severe anxiety without AEs, minimal/mild anxiety without AEs, and patients who experienced AEs regardless of anxiety severity. The green band represents the interquartile exposure interval associated with favorable therapeutic response and tolerability within this cohort (27.85–57.91 ng/mL), while the blue band corresponds to the broader range defined by the 10th to 90th percentiles (P10-P90; 14.80–103.91 ng/mL). These findings should be interpreted as exploratory and hypothesis-generating rather than as a validated therapeutic target range.

**FIGURE 7:**
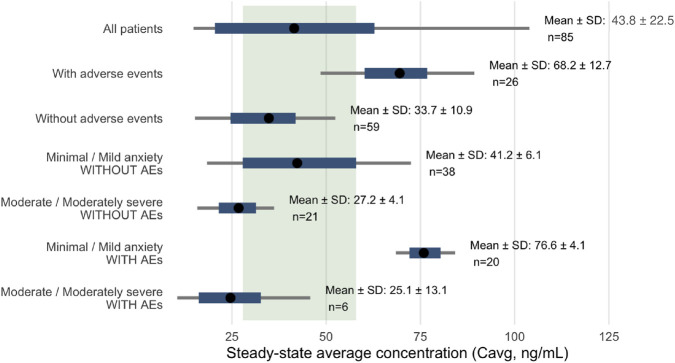
Distribution of steady-state average concentrations (Cavg) across clinically defined subgroups. Horizontal plots represent mean ± standard deviation (blue bars) and distribution ranges for each subgroup, including all patients, patients with and without AEs, and stratified groups according to anxiety severity (minimal/mild vs. moderate/moderately severe) and AE status. Black dots indicate mean values. The shaded green area represents the proposed exposure range associated with a favorable balance between efficacy and safety (27.85–57.91 ng/mL). Patients with AEs show a shift toward higher concentrations, particularly in the minimal/mild anxiety subgroup, whereas patients with greater anxiety severity tend to exhibit lower exposure. The variability across groups highlights the impact of pharmacokinetic differences on clinical outcomes.

An exploratory analysis was conducted to identify an exposure range associated with favorable clinical outcomes. This range was derived from individual model-based exposure estimates restricted to patients with favorable clinical outcomes. Patients with minimal or mild anxiety and no AEs exhibited an interquartile range of steady-state average concentrations (Cavg) between 27.85 and 57.91 ng/mL. When focusing on steady-state exposure and minimizing the influence of extreme peak concentrations, the interquartile range of Cavg (27.85–57.91 ng/mL) was considered the most clinically relevant exposure window, as it reflects typical systemic exposure in patients with favorable response and absence of AEs. Additionally, the broader exposure range defined by the 10th to 90th percentiles (P10-P90) was 14.80 to 103.91 ng/mL, capturing most of the observed interindividual variability. To further contextualize the proposed exposure range, observed sertraline concentrations were visualized across the dosing interval (Figure 5). Each patient contributed between one and three plasma samples, reflecting the sparse sampling design of the study. The distribution of observed concentrations shows that a substantial proportion of values fall within the proposed exposure range (27.85–57.91 ng/mL), while values below and above this interval are also observed. The shaded region representing the 5th to 95th percentile range illustrates the extent of interindividual variability in systemic exposure. Concentrations below the interquartile range were predominantly observed in patients with moderate/moderately severe anxiety, whereas higher concentrations were observed in patients with minimal/mild anxiety and in those with AEs, particularly in patients with lower clearance. These findings are summarized in Table 5 and Figure 6. To further characterize the distribution of steady-state exposure across clinically defined subgroups, an additional visualization incorporating median values, interquartile ranges (IQR), and P10-P90 intervals was constructed (Figure 7). While Figure 6 provides a global distribution of Cavg, this representation allows a more structured comparison across subgroups. Patients with minimal/mild anxiety without AEs exhibited Cavg values predominantly within the proposed exposure range (27.85–57.91 ng/mL), whereas those with AEs showed a clear shift toward higher concentrations. The wider P10-P90 intervals further illustrate substantial interindividual variability, with some patients reaching concentrations above 100 ng/mL despite moderate average exposure values. A detailed summary of distributional metrics for steady-state average concentrations (Cavg), including mean, median, interquartile range (IQR), and P10-P90 intervals across all clinical subgroups, is provided in Supplementary Table S1. This table complements the graphical representations by providing precise numerical values and further supports the observed differences in systemic exposure between patients with and without AEs, as well as across anxiety severity categories.

**TABLE 5:** Sertraline exposure ranges and their clinical interpretation.

Exposure range	Concentration interval (ng/mL)	Clinical criterion	Interpretation
Exposure range associated with favorable response (IQR)	27.85–57.91	Minimal/mild anxiety (*GAD-7*) without AEs	Associated with a favorable balance between efficacy and safety
Extended range (P10-P90)	14.80–103.91	Overall cohort distribution	Reflects interindividual variability in drug exposure

IQR: interquartile range; P10-P90: 10th to 90th percentiles; GAD-7: *Generalized Anxiety Disorder-7*; AEs: adverse events; Exposure ranges were derived from steady-state average concentrations (Cavg) estimated using the population pharmacokinetic model; The exposure range associated with favorable response should be considered exploratory and requires validation in independent studies.

## Discussion

### Overall interpretation of findings

In this study, PKpop profile of sertraline was characterized in pediatric patients with anxiety disorders, and its relationship with clinical response and AEs was evaluated. Overall, the results demonstrated substantial interindividual variability in systemic exposure, along with consistent associations between PK parameters and clinical outcomes. Notably, clear patterns emerged linking sertraline exposure to both anxiety severity and the probability of developing AEs, supporting the clinical relevance of an exposure-response framework. From a clinical perspective, approximately one-third of patients experienced at least one AEs during treatment, representing a clinically meaningful frequency in pediatric settings. Although no statistically significant differences were observed in variables such as age, sex, or dose, trends were identified in anthropometric parameters, particularly body weight and body mass index (BMI), with higher values observed in patients who developed AEs. This observation is consistent with previous reports suggesting that exposure to selective serotonin reuptake inhibitors (SSRIs) may be associated with changes in body composition, including increases in BMI and fat mass, which may indirectly influence drug pharmacokinetics (Calarge et al., 2017).

### Pharmacokinetic implications and biological plausibility

The PKpop model that best described the data was a two-compartment model with first-order absorption and a lag time, consistent with the known physicochemical properties of sertraline. This structural behavior suggests extensive tissue distribution, likely driven by its high lipophilicity and strong plasma protein binding. Previous studies have shown that SSRIs exhibit distribution beyond the vascular compartment, facilitated by their lipophilic nature (DeVane, 1992; Hiemke et al., 2018). In particular, these compounds demonstrate affinity for adipose tissue, which contributes to increased volume of distribution and prolonged residence time in the body (Han et al., 2007; Cheymol, 2000). In this context, the findings of the present study support the biological plausibility of the two-compartment model and highlight the contribution of peripheral compartments to drug disposition. Although sertraline is highly lipophilic and extensively distributed, the estimated apparent central and peripheral volumes of distribution were numerically large and should be interpreted cautiously. In the absence of intravenous data, oral bioavailability could not be separated from distribution volume; therefore, V1/F and V2/F represent apparent rather than physiological volumes. The additional diagnostic evaluation supports this interpretation: CL/F, the main exposure-driving parameter, showed low eta-shrinkage, whereas V2/F and Q/F showed higher shrinkage, suggesting limited individual-level identifiability of peripheral distribution parameters under the sparse sampling design. Thus, while the model remains useful for estimating systemic exposure and exploring the exploratory exposure-response associations, distribution-related parameters should be interpreted primarily as model-based descriptors rather than direct physiological estimates. Although sparse pediatric sampling may limit full identifiability of some structural parameters, the two-compartment model consistently outperformed simpler one-compartment alternatives across statistical, diagnostic, and predictive criteria. Therefore, the selected model should be interpreted as the structural model that best described the observed concentration-time data within the constraints of the available sampling design.

Interindividual variability in pharmacokinetic parameters was moderate and consistent with previous reports in pediatric populations receiving SSRIs. The identification of body weight and BMI as significant covariates affecting apparent clearance (CL/F) and central volume of distribution (V1/F) suggests that body composition plays a relevant role in sertraline pharmacokinetics. This is consistent with evidence indicating that differences in fat and lean mass proportions can influence the distribution and elimination of lipophilic drugs, with direct implications for systemic exposure. An additional factor that may contribute to the observed interindividual variability in sertraline exposure is pharmacogenetic variability involving cytochrome P450 enzymes, particularly *CYP2C19, CYP2D6*, and *CYP2B6*. Previous studies have demonstrated that *CYP2C19* phenotype may significantly influence sertraline clearance and systemic exposure in pediatric populations. However, pharmacogenetic analyses were not performed in the present study; therefore, the contribution of specific metabolic phenotypes could not be directly evaluated. Consequently, interpretations regarding CYP-mediated variability should be considered biologically plausible but exploratory rather than mechanistically confirmed within this cohort.

### Exposure-response relationship: efficacy and safety

One of the most clinically relevant findings of this study was the association between systemic exposure to sertraline and clinical outcomes. Patients with lower apparent clearance exhibited higher systemic exposure, which translated into an increased likelihood of AEs. This observation is consistent with fundamental PK principles, whereby reduced clearance leads to increased exposure and a higher risk of drug-related toxicity. In the context of SSRIs, elevated plasma concentrations have been associated with a higher incidence of AEs, particularly in vulnerable populations such as pediatric patients. Additionally, patients with AEs exhibited not only higher systemic exposure but also reduced variability in Cavg, indicating a clustering of concentrations at higher levels. This pattern may reflect a subgroup with consistently lower clearance, resulting in sustained elevated exposure and increased susceptibility to AEs. The presence of concentrations exceeding 100 ng/mL in some individuals, as observed in Figure 7, reflects peak exposure variability and should be interpreted in the context of the difference between average exposure (Cavg) and transient peak concentrations. Previous studies have demonstrated that variability in antidepressant plasma levels is linked to differences in tolerability, with higher concentrations associated with increased AEs (Wyska, 2019; Lundmark et al., 2000). These findings are in agreement with the present study, where increased sertraline exposure was associated with a higher probability of AEs.

Furthermore, logistic regression analysis demonstrated that both lower clearance and higher Cmax values were associated with a quantifiable increase in the risk of AEs, reinforcing the clinical relevance of individual pharmacokinetic variability. Conversely, in terms of therapeutic response, patients with greater anxiety severity exhibited higher apparent clearance and, consequently, substantially lower Cavg. This finding suggests that insufficient systemic exposure may limit the therapeutic efficacy of sertraline, consistent with exposure-response relationship principles indicating that clinical response depends on achieving adequate drug concentrations to sustain serotonin transporter inhibition. Taken together, these results suggest that systemic exposure may contribute to differences in both efficacy and safety outcomes in sertraline-treated pediatric patients.

### Clinical implications and proposed exposure range

Given that a well-defined therapeutic range for sertraline in pediatric populations remains lacking and that previously reported ranges are broad and variable, an exploratory analysis was conducted to identify an exposure range associated with an optimal balance between efficacy and safety. Previous studies have highlighted the difficulty of defining a universal therapeutic range for sertraline due to substantial interindividual variability in both exposure and clinical response. For example, Hiemke et al. (2018), reported wide therapeutic ranges (10–150 ng/mL), reflecting this heterogeneity. Similarly, Tini et al. (2022), identified an interquartile range associated with clinical response in pediatric obsessive-compulsive disorder; however, that study did not simultaneously evaluate the relationship with AEs, limiting its clinical applicability.

In the present cohort, patients with minimal or mild anxiety and no AEs exhibited an interquartile range of steady-state concentrations (Cavg) between 27.85 and 57.91 ng/mL, which may represent a clinically favorable exposure window in this population. From a clinical perspective, and acknowledging the exploratory nature of the present analyses as well as the potential influence of non-representative peak concentrations, the exposure interval of 27.85–57.91 ng/mL may represent a preliminary exposure range associated with the balance between therapeutic response and tolerability observed in this cohort. In contrast, the broader P10-P90 interval (14.80–103.91 ng/mL) encompassed most of the observed interindividual variability in systemic exposure. These findings should be interpreted as hypothesis-generating and require prospective validation in future PK/PD studies before being considered for clinical application. Importantly, concentrations below the interquartile range were primarily observed in patients with moderate/moderately severe anxiety, whereas concentrations above this range were associated with an increased frequency of AEs, particularly in patients with lower clearance. These findings suggest the existence of a therapeutic window in which the balance between efficacy and safety is optimized (Reis et al., 2009). From a clinical perspective, these results support the potential role of therapeutic drug monitoring (TDM) as a tool for dose individualization in pediatric patients, particularly in those with suboptimal response or increased susceptibility to AEs. The applicability of this exposure interval to broader pediatric populations remains uncertain because the present cohort was recruited from two centers in Mexico and did not include pharmacogenetic characterization. Prospective validation in geographically and ethnically diverse populations will be necessary to determine the external validity of these findings.

### Strengths of the study

This study presents several strengths that support the robustness and clinical relevance of its findings. First, the use of a population pharmacokinetic (PKpop) approach based on nonlinear mixed-effects modeling allowed a comprehensive characterization of interindividual variability in sertraline disposition in a pediatric population, despite the implementation of a sparse sampling design. This approach is particularly suitable for vulnerable populations such as pediatric patients, where ethical and logistical constraints limit the feasibility of intensive sampling. Furthermore, the study integrates pharmacokinetic analysis with clinically meaningful outcomes, including both therapeutic efficacy and safety, enabling the characterization of an exposure-response relationship with direct clinical implications. This exposure-response analysis represents a significant strength, as it extends beyond traditional descriptive analyses and contributes to a more mechanistic understanding of the determinants of treatment response.

Another important strength lies in the rigorous validation of the pharmacokinetic model, which included goodness-of-fit diagnostics, visual predictive checks, and bootstrap analysis. These approaches support the stability, reliability, and predictive performance of the developed model. In addition, the identification of clinically plausible covariates, such as body weight and body mass index, provides further insight into factors contributing to pharmacokinetic variability in this population. Finally, the study proposes, in an exploratory manner, an exposure range associated with a favorable balance between efficacy and safety, derived from real-world clinical data. This finding represents an innovative contribution with potential applicability in clinical practice, particularly in the context of therapeutic drug monitoring and dose individualization in pediatric patients with anxiety disorders.

### Limitations of the study

Several limitations should be considered when interpreting the findings of this study. First, although the sample size was adequate for population pharmacokinetic modeling, it may limit the generalizability of the results to other pediatric populations with different clinical, genetic, or environmental characteristics. Therefore, extrapolation of these findings to other settings should be undertaken with caution. Second, the observational design of the study, while reflecting real-world clinical practice and enhancing external validity, limits the ability to establish definitive causal relationships between sertraline exposure and clinical outcomes. Accordingly, the associations identified between pharmacokinetic parameters, therapeutic response, and AEs should be interpreted as associative rather than causal.

Another important limitation is the absence of pharmacogenetic characterization. Although variability in *CYP2C19, CYP2D6*, and *CYP2B6* activity is recognized as an important determinant of sertraline pharmacokinetics, genetic data were not collected in the present study. Consequently, the mechanisms underlying the observed interindividual variability in systemic exposure could not be fully characterized. Future studies integrating pharmacogenetic information with population PK/PD approaches may help better explain differences in therapeutic response and susceptibility to AEs.

Although the sparse sampling strategy was appropriate for a pediatric population, it likely reduced the precision of distribution-related parameters, particularly V2/F and Q/F. This was supported by higher eta-shrinkage for V2/F and Q/F, whereas CL/F showed low shrinkage, supporting the reliability of the main exposure-driving parameter used in the exposure-response analyses. Eta-correlation analysis did not indicate critical collinearity between peripheral distribution parameters; however, the moderate correlation between ETA_CL and ETA_V1 suggests that apparent structural parameters should still be interpreted cautiously. The relatively wide confidence interval associated with the estimated elimination half-life further suggests uncertainty in the characterization of terminal disposition parameters. Consequently, half-life estimates should be interpreted cautiously and primarily as model-derived descriptors rather than precisely identified physiological parameters. Although the final two-compartment model demonstrated superior statistical and diagnostic performance compared with simpler structural alternatives, the sparse sampling design limits the extent to which physiological compartment behavior can be definitively characterized. Therefore, the structural model should primarily be interpreted as a robust empirical representation of sertraline disposition in this cohort rather than as definitive evidence of fully identifiable physiological compartments.

Although AEs were systematically solicited during scheduled follow-up visits and graded according to CTCAE criteria, formal causality assessment was not performed. Furthermore, while all reported AEs occurred within the first month of treatment, the precise time-to-onset of individual events relative to pharmacokinetic exposure was not incorporated into the analyses. Moreover, although additional multivariable analyses adjusting for age, BMI, and dose were performed, residual confounding cannot be completely excluded. Other potentially relevant variables, including objective adherence measures, treatment duration variability, and baseline clinical characteristics, were not simultaneously incorporated into the adjusted models. Therefore, the observed exposure-response associations should still be interpreted as exploratory and hypothesis-generating rather than definitive evidence of independent causal effects. In addition, the available dataset was not considered sufficient to support development of a formal PK/PD model. Therefore, exposure-response analyses were limited to exploratory regression-based approaches using model-derived exposure metrics. This may limit the ability to fully characterize the dynamic relationship between systemic exposure and clinical outcomes. Similarly, the categorization of PK parameters, such as apparent clearance, may result in loss of information compared with continuous modeling approaches. Finally, the proposed exposure range was derived from an exploratory analysis integrating efficacy and safety outcomes within this specific cohort. Although this approach provides clinically relevant insights, its applicability should be considered preliminary and requires validation in independent studies with larger sample sizes, prospective designs, and diverse clinical settings before being adopted as a therapeutic reference in clinical practice.

### Future directions

The findings of the present study open several avenues for future research aimed at advancing the understanding of the exposure-response relationship of sertraline in pediatric populations. First, validation of these results in independent cohorts with larger sample sizes and greater clinical, genetic, and geographic diversity is essential to confirm the reproducibility and generalizability of both the pharmacokinetic parameters and the exposure range associated with favorable clinical outcomes. Future studies should also incorporate prospective designs with more PK/PD modeling approaches, including mechanistic or semi-mechanistic models, to better characterize the relationship between systemic exposure, therapeutic response, and AEs. In this context, the implementation of longitudinal models capturing the temporal evolution of anxiety severity could provide a more detailed understanding of treatment dynamics. Another important research direction involves the integration of pharmacogenetic data into population PK/PD models, particularly variants affecting *CYP2C19, CYP2D6,* and *CYP2B6*. Given the established influence of these enzymes on sertraline metabolism, incorporation of pharmacogenetic characterization may help explain a substantial proportion of the interindividual variability observed in systemic exposure, therapeutic response, and AEs. Such approaches could facilitate the development of precision medicine strategies and individualized dosing algorithms for pediatric patients with anxiety disorders. Future studies should evaluate the reproducibility of the proposed exposure interval in larger multicenter cohorts representing diverse ethnic and pharmacogenetic backgrounds.

## Conclusion

The results of this study demonstrate a significant association between sertraline pharmacokinetic variability and clinical outcomes in pediatric patients with anxiety disorders. Systemic exposure to the drug was consistently associated with both therapeutic response and the occurrence of AEs, with lower apparent clearance linked to an increased risk of AEs, whereas higher clearance appeared to compromise clinical efficacy by reducing systemic exposure. An exposure range of approximately 28–58 ng/mL (Cavg) was identified as being associated with optimal clinical outcomes, supporting its potential role as a preliminary exposure range for therapeutic drug monitoring. Although this range should be considered exploratory, it provides clinically relevant insight for treatment optimization. Nevertheless, the absence of pharmacogenetic characterization limits mechanistic interpretation of the observed pharmacokinetic variability and should be considered when interpreting these findings. Overall, these findings highlight the importance of accounting for individual pharmacokinetic variability in therapeutic decision-making and support the potential role of therapeutic drug monitoring as a strategy for dose individualization in pediatric patients receiving sertraline. Such an approach may improve treatment efficacy while reducing the incidence of AEs, contributing to more precise and safer management of anxiety disorders in this population.

## Data Availability

The original contributions presented in the study are included in the article/Supplementary Material, further inquiries can be directed to the corresponding authors.
