# Supplementary material for: Exposure-response relationship of sertraline in pediatric patients with anxiety disorders: a population pharmacokinetic analysis
Source: Front Pharmacol. 2026 Jun 24;17:1862275. doi: 10.3389/fphar.2026.1862275 (PMC13342236; doi:10.3389/fphar.2026.1862275)
Supplement: Supplementary file 1 [file DataSheet1.docx]

**Supplementary Table S1. Comparison of Evaluated Structural Pharmacokinetic Models**

| Model | Structural Characteristics | OFV | ΔOFV vs Previous | AIC | BIC | Residual Error | GOF Diagnostics | VPC Performance | Bootstrap Stability | Interpretation |
| --- | --- | --- | --- | --- | --- | --- | --- | --- | --- | --- |
| Model 1 | One-compartment, first-order absorption | 2317 | - | 1298 | 1332 | Proportional | Systematic underprediction at late time points | Poor | Moderate instability | Inadequate description of distribution phase |
| Model 2 | One-compartment + Tlag | 2289 | -28 | 1241 | 1284 | Combined | Slight improvement during absorption phase | Acceptable | Moderate | Improved absorption fit but persistent bias |
| Model 3 | Two-compartment, first-order absorption | 2248 | -41 | 1087 | 1136 | Combined | Improved residual distribution | Good | Acceptable | Better characterization of concentration decline |
| Model 4 (Final) | Two-compartment + Tlag | 2216 | -32 | 982 | 1044 | Combined | Randomly distributed residuals without systematic trends | Good | Stable across bootstrap replicates | Best overall description of sparse pediatric PK data |

- Structural models were evaluated sequentially using nonlinear mixed-effects modeling implemented in Monolix®.
- Model selection considered statistical criteria (OFV, AIC, BIC), goodness-of-fit diagnostics, visual predictive checks (VPC), bootstrap robustness, and biological plausibility.
- The final model was selected because it provided the best balance between statistical performance and clinical interpretability.
- Due to sparse pediatric sampling (1–3 samples per patient), the final model should be interpreted as the model that best described the available data rather than definitive evidence of fully identifiable physiological compartments.
- Despite these limitations, the final model generated clinically useful exposure estimates (Cavg, CL/F, Cmax) that supported the exposure-response analyses involving therapeutic response and adverse events.

**Supplementary Table S2.-** **Models adjusted for age, body mass index, and sertraline dose.**

| **Outcome** | **Predictor** | **Adjusted OR** | **95% CI** | **p** |
| --- | --- | --- | --- | --- |
| AEs | Cavg | 1.664 | 1.061-2.607 | 0.026 |
| AEs | Cmax | 1.456 | 1.103-1.923 | 0.008 |
| AEs | CL/F | 0.658 | 0.529-0.819 | <0.001 |
| Moderate/Severe Anxiety | Cavg | 0.954 | 0.925-0.983 | 0.002 |
| Moderate/Severe Anxiety | CL/F | 1.109 | 1.049-1.172 | <0.001 |

Adjusted analyses were performed as sensitivity analyses to evaluate the robustness of the observed exposure-response associations. Odds ratios are expressed per unit increase in the corresponding pharmacokinetic parameter. Models were adjusted for age, body mass index (BMI), and sertraline dose.

| Group | n | Mean Cavg (ng/mL) | Median (P50) | IQR (P25-P75) | P10-P90 |
| --- | --- | --- | --- | --- | --- |
| All patients | 85 | 43.8 ± 22.5 | 41.5 | 20.5 -62.8 | 14.8 - 103.9 |
| With adverse events | 26 | 68.2 ± 12.7 | 69.5 | 60.2 -76.8 | 48.5 - 89.3 |
| Without adverse events | 59 | 33.7 ± 10.9 | 34.8 | 24.6 - 41.9 | 15.2 - 52.4 |
| Minimal / Mild anxiety WITHOUT AEs | 38 | 41.2 ± 6.1 | 42.3 | 27.85 - 57.91 | 18.4 - 72.5 |
| Moderate / Moderate-severe WITHOUT AEs | 21 | 27.2 ± 4.1 | 26.8 | 21.5 - 31.4 | 15.8 - 36.2 |
| Minimal / Mild anxiety WITH AEs | 20 | 76.6 ± 4.1 | 75.9 | 72.1 - 80.3 | 68.5 - 84.2 |
| Moderate / Moderate-severe WITH AEs | 6 | 25.1 ± 13.1 | 24.6 | 16.2 - 32.7 | 10.5 - 45.8 |

**Supplementary Table S3.** Summary of steady-state exposure (Cavg) metrics across clinical subgroups, including mean, median, interquartile range, and percentile distribution.


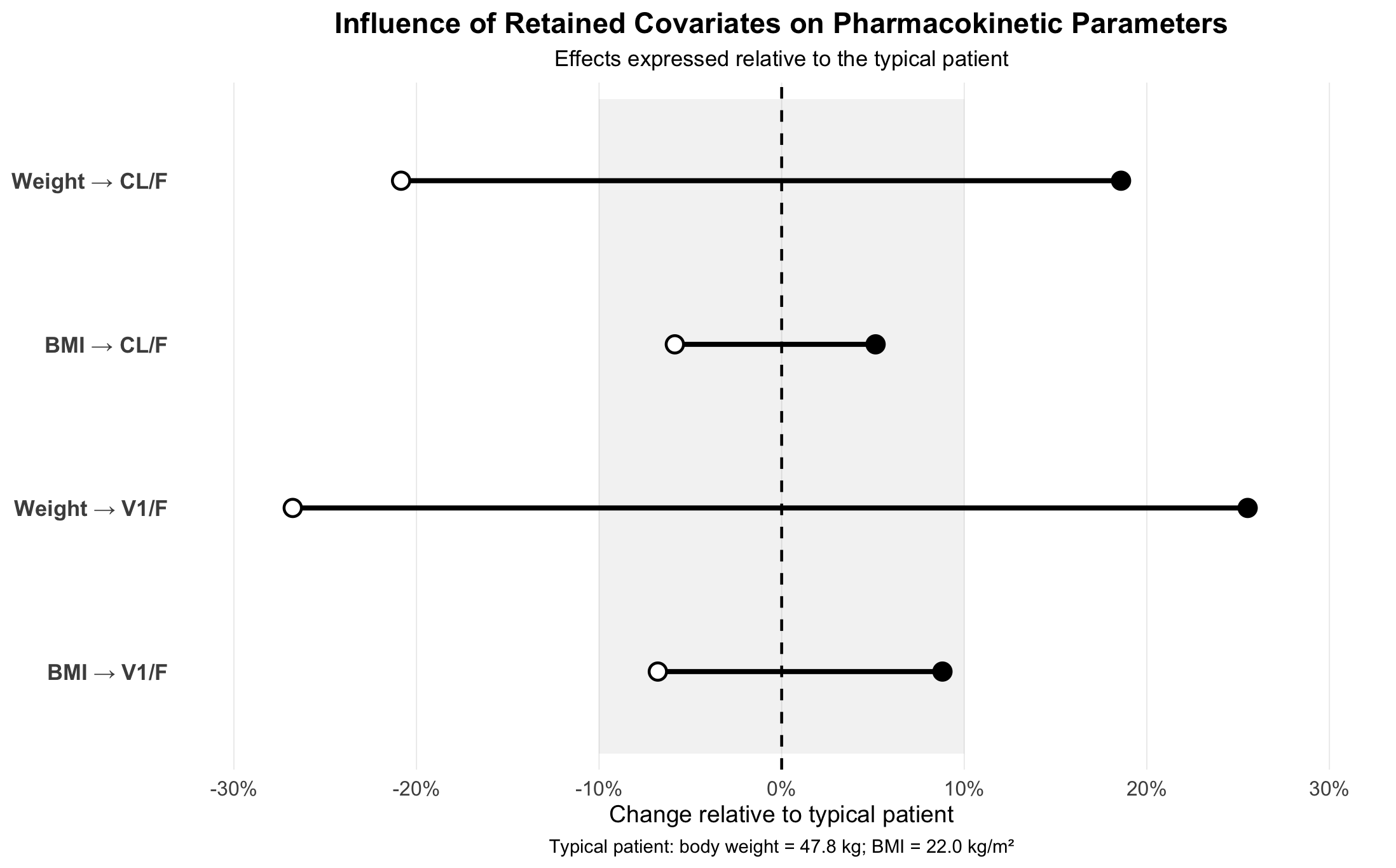


**Supplementary Figure S1. Influence of retained covariates on pharmacokinetic parameters in the final population pharmacokinetic model of sertraline.** Forest plot illustrating the magnitude and direction of the effects of the retained covariates (body weight and body mass index [BMI]) on apparent clearance (CL/F) and apparent central volume of distribution (V1/F) in the final population pharmacokinetic model. Effects are expressed as percentage change relative to the typical patient (body weight = 47.8 kg; BMI = 22.0 kg/m²). Positive values indicate an increase in the parameter estimate, whereas negative values indicate a decrease relative to the typical patient. Body weight and BMI were retained as significant predictors of pharmacokinetic variability and contributed to reducing unexplained interindividual variability in the final model.
